# Tumor Suppressor miR-613 Alleviates Non-Small Cell Lung Cancer Cell via Repressing M2 Macrophage Polarization

**DOI:** 10.1155/2023/2311231

**Published:** 2023-02-16

**Authors:** Mingjun Yang, Wen Zhou, Mingming Xu, Xiao Han, Yanyan Shi, Min Shi, Zhipeng Wang

**Affiliations:** ^1^Department of Cardiothoracic Surgery, Affiliated Hospital of Nantong University, No. 20, Xisi Road, Nantong, Jiangsu 226001, China; ^2^Department of Thoracic Surgery, Haimen People's Hospital, No. 1201, Beijing Road, Nantong, Jiangsu 226001, China

## Abstract

**Background:**

Non-small cell lung cancer (NSCLC) is a crucial crux of cancer-related death, and M2 macrophage polarization facilitates NSCLC development. MicroRNA-613 (miR-613) is a tumor suppressor. This research aimed to clarify the miR-613 function in NSCLC and its impact on M2 macrophage polarization. *Methods. *miR-613 expressions in NSCLC tissues and cells were evaluated using quantitative real-time PCR. For miR-613 function in NSCLC, cell proliferation analysis, cell counting kit-8, flow cytometry, western blot, transwell, and wound-healing were conducted. Meanwhile, the miR-613 impact on M2 macrophage polarization was assessed by the NSCLC models. *Results. *miR-613 was lessened in NSCLC cells and tissues. It was corroborated that miR-613 overexpression retrained NSCLC cell proliferation, invasion, and migration but facilitated cell apoptosis. Moreover, miR-613 overexpression restrained NSCLC development by repressing M2 macrophage polarization.

**Conclusion:**

Tumor suppressor miR-613 ameliorated NSCLC by restraining M2 macrophage polarization.

## 1. Introduction

Lung cancer is a malignant tumor worldwide with high frequency in morbidity and mortality [1]. Nearly, 85% of lung cancer is non-small-cell lung cancer (NSCLC) [2, 3]. Although multiple progress has been achieved in NSCLC treatment, the effect is unsatisfactory [4, 5]. Thus, there is an urgent need to better understand NSCLC pathogenesis and identify novel therapeutic targets to alleviate NSCLC.

In search for more effective intervention methods for NSCLC, the importance of targeting the tumor microenvironment (TME) has gradually attracted wide attention [6, 7]. TME forms of cancer and stromal cells containing macrophages, and endothelial cells [8, 9]. As one of the pivotal components of TME, tumor-associated macrophages (TAMs) are major coordinators of immune cells [10]. TAMs mainly include M1 and M2, and M2 macrophages accelerate tumorigenesis [11, 12]. Crucially, accumulated studies demonstrate that M2 macrophages drive proliferation, migration, invasion, and other malignant phenotypes of NSCLC, thereby aggravating NSCLC [13, 14]. Nevertheless, the definite mechanism of M2 macrophages facilitating the NSCLC occurrence is not fully clarified.

MicroRNAs (miRNAs) are highly conserved small noncoding RNAs [15], exert pivotal functions in various human malignancies, and are promising targets for tumor therapy [16, 17]. Recently, increasing miRNAs have been confirmed to mediate the NSCLC process. For instance, miR-130a mediates macrophage polarization in NSCLC, and miR-130a loss is interrelated to NSCLC-poor prognosis and increased tumor stage [18]; miR-4319 knockdown accelerates NSCLC tumor progression by accelerating M2 macrophage polarization, which might supply promising strategies for NSCLC treatment [19].

miR-613 is a widely functional miRNA in various tumors. It has been reported that miR-613 slows down the process of cervical squamous cell cancer by targeting LETM1 [20]. miR-613 can regulate gastric cancer progression by regulating reactive oxygen species (ROS) production, glutathione content (GSH), and superoxide dismutase (SOD) activity [21]. miR-613 represses cell migration and invasion in esophageal squamous cell carcinoma via mediating G6PD to inactivate the STAT3 signaling pathway [22]. miR-613 induces NSCLC cell sensitivity to cisplatin by targeting GJA1 [23]. However, it remains unknown whether miR-613 mediates M2 macrophage polarization in NSCLC.

The current research confirmed the low expression of miR-613 in NSCLC tissues and cells. On this basis, this study attempted to further investigate whether miR-613 participated in the NSCLC regulation by mediating M2 macrophage polarization.

## 2. Materials and Methods

### 2.1. Samples

NSCLC and adjacent normal tissues (*n* = 20 for each) were gathered from patients at Affiliated Hospital of Nantong University and stored with liquid nitrogen for the follow-up research. Informed consent was signed by all patients before performing surgery. The present research was permitted by the Ethics Committee.

### 2.2. Cell Culture

Normal human lung cell lines HLF-A was from Procell (Wuhan, China), NSCLC cells HCC827, H1299, H1650, and A549 were from the American Type Culture Collection (ATCC, Maryland, USA), and THP-1 cells were from ATCC.

HLF-A cells were put in minimum essential medium (Thermo Fisher Scientific, Waltham, Massachusetts, USA) containing 1% penicillin, 10% FBS, and streptomycin (Gibco, New York, USA). NSCLC cells HCC827, H1299, and H1650 were put in RPMI-1640 with 10% FBS. THP-1 cells were put in RPMI-1640 with 10% FBS and 0.05 mM 2-mercaptoethanol. A549 cells were grown in ATCC-formulated F-12K (ATCC) containing 10% FBS. All cells were cultivated at 37°C, 5% CO_2_.

For macrophage induction, THP-1 cells were differentiated into macrophages by treatment with phorbol 12-myristate 13-acetate (150 nM, Sigma-Aldrich, St Louis, USA) for 1 d [24]. The cells were developed with 20 ng/mL IL-4 (MedChemExpress, New Jersey, USA) for 48 h to achieve M2 macrophages [13].

### 2.3. Cell Transfection

miR-613 mimic and NC-mimic were obtained from Ribobio (Guangzhou, China). H1650 cells and M2 macrophages (1 × 10^6^) were placed in six-hole plates and cultured overnight, followed by cell transfection with miR-613 mimic or NC-mimic after 24 h using Lipofectamine 2000 following the manufacturer's protocols.

### 2.4. Co-Culture System

Transwell inserts with 0.4 *μ*M aperture from Corning (New York, USA) were applied as a co-culture system. THP-1 cells (2 × 10^5^) were placed in Transwell inserts and handled with 20 ng/mL IL-4 for 2 d to differentiate to M2 macrophages, and the miR-613 mimic was transfected into M2 macrophages. Afterward, the upper chambers were transferred into Petri dishes with H1650 cells and co-cultured for 24 h.

### 2.5. RNA Extraction

Total RNA was extracted in a TRIzol reagent (Beyotime, Shanghai, China) as per standard procedure, and then, RNA content and quality were evaluated via detecting optical density (A260, A280, and A230).

### 2.6. Quantitative Real-Time PCR

cDNA was synthesized with A cDNA synthesis kit (Thermo Fisher Scientific) and one step PrimeScript miRNA cDNA synthesis kit (TaKaRa, Dalian, China), referring to manufacturers' protocol. Real-time was run on ABI 7300 real-time qPCR system (ABI company) with SYBR green PCR kits (Thermo Fisher Scientific). For the miR-613 expression analysis, qRT-PCR was conducted with TaqMan microRNA assay. U6 and *β*-actin were internal references. The relative level was tested through 2^−∆∆CT^ [25].  Table 1 lists all primer sequences.

### 2.7. Cell Counting Kit-8 (CCK-8) Assay

H1650 cells treated differently (1 × 10^3^) were inoculated in plates with 96 holes. The plates were placed in the incubator and incubated for 24 h, 48 h, or 72 h (at 37°C, 5% CO_2_). Then, the cells were handled for 1.5 h at 37 °C using 10 *μ*L CCK-8 solution (Beyotime). Afterward, cell viability was assessed with a microplate reader (Bio-Rad, California, USA).

### 2.8. Cell Proliferation

The proliferation of H1650 cells was determined using EdU Cell Proliferation Kits (Ribobio) following a standard procedure. H1650 cells (1 × 10^3^) were placed in 96-well plates and cultured for one day. EdU (50 *μ*M) was incubated with cells for two hours. Cells were then fastened with 4% paraformaldehyde (Solarbio, Beijing) and dyed by Hoechst 33342 and an Apollo reaction cocktail. The images were obtained by fluorescence microscopy (Olympus, Tokyo, Japan). The percentage of positive cells was counted and calculated using ImageJ2X (Rawak Software Inc.).

### 2.9. Flow Cytometry

The apoptosis condition of H1650 cells was assessed via FITC Annexin V Apoptosis Detection Kit I (BD Biosciences, New Jersey, USA). After H1650 cells were harvested, the cells were resuspended, handled for 20 min with FITC Annexin V at 37°C, and further handled for 20 min with propidium iodide (PI) in the dark. H1650 cell apoptosis was tested with a FACSCalibur Flow Cytometer (BD Biosciences).

### 2.10. Western Blot

RIPA lysis buffer (Solarbio) was adopted to extract total proteins following protocol. After protein content was tested by BCA assay kits (Abcam), the same amount of protein was segregated with SDS-PAGE (Thermo Fisher Scientific) before transferring it onto PVDF membranes (Millipore, Massachusetts). Then, membranes were fostered for one hour with 5% skimmed milk and further treated at 4°C overnight with primary antibodies containing anti-Bax (Abcam, 1 : 1000, ab32503), anti-Bcl2 (Abcam, 1 : 1000, ab32124), anti-*β*-actin (Abcam, 1 *µ*g/mL, ab8226), and anti-cleaved-caspase-3 (Abcam, 1 : 500, ab32042). Subsequently, these membranes were incubated with secondary antibodies (Abcam). Proteins were visualized using an electrochemiluminescence system (Solarbio) and were evaluated by ImageJ2X (Rawak Software Inc.).

### 2.11. Wound-Healing Assay

H1650 cells were fostered to 50% confluence in six-hole plates; the 1% double-antibody serum-free medium was replaced. Then, the plate surface was lightly scratched by 200 *μ*L pipette tips and put into a 37°C 5% CO_2_ incubator for culture. After scratching with an inverted optical microscope (Olympus), H1650 cell migration was monitored at 0 h and 48 h. The migration area was measured using ImageJ2X (Rawak Software Inc.), and the mobility was calculated.

### 2.12. Transwell Analysis

For cell migration analysis, H1650 cells (5 × 10^4^) were suspended in a FBS-free medium (100 *μ*L) and transferred onto an upper chamber (Corning, Cambridge) with noncoated membranes. Lower chambers were filled with standard medium (Thermo Fisher Scientific). After incubating for 24 h, the cells on the membrane upper surface were removed and stained cells on the membrane lower surface with crystal violet (0.1%, Solarbio).

For cell invasion analysis, Matrigel chambers were conducted. After H1650 cells were harvested, they were resuspended in a serum-free medium and transferred to hydrated matrix chambers (50 *μ*L). The bottom chambers were put in RPMI-1640 (500 *μ*L) containing 10% FBS. On the next day, cells on the upper surface were scraped and stained infiltrating cells on the lower surface by 0.1% crystal violet (Solarbio). All cells were counted with 3–5 random fields. Then, we use ImageJ2X (Rawak Software Inc.) to quantify.

### 2.13. Enzyme-Linked Immunosorbent Assay

IL-10 and TGF-*β* levels in M2 macrophage culture supernatant were tested with enzyme-linked immunosorbent assay with IL-10 ELISA Kit (Thermo Fisher Scientific) and TGF-*β* ELISA Kit (Spbio, Wuhan, China), referring to manufacturer's instructions.

### 2.14. *In Vivo* Assay

C57BL/6 mice (5 weeks old) were from Vital River Laboratory Animal Technology (Beijing, China).

Macrophages with different treatments were co-cultured with H1650 cells. H1650 cells were grouped: H1650+M^NCmimic^, H1650+M^IL-4^^+^^NCmimic^ and H1650+M^IL-4^^+^^miR-613mimic^. The mice were subcutaneously injected with the abovementioned H1650 cells (5 × 10^5^) [13]. The tumor volumes were tested with a vernier caliper. Three weeks after inoculation, sacrificed all mice with an intraperitoneal injection of pentobarbital sodium (100 mg/kg), gathered and weighed tumor tissues for subsequent studies.

### 2.15. TUNEL and Ki-67 Staining

TUNEL kit (Beyotime) was applied for TUNEL staining following instructions. Xenograft tissues were fastened in 4% paraformaldehyde, embedded in paraffin, and deparaffinized and rehydrated. Followed by the antigen retrieval, sections (5 *μ*m) were incubated for 1.5 h with a 50 *μ*L TUNEL mix at 37°C, washed, and DAPI (2 *µ*g/mL) was added for nuclei staining, followed by washing using PBS and observing under a fluorescence microscope (OLYMPUS).

For Ki-67 staining, the abovementioned sections were handled by anti-Ki-67 (Abcam, 1 *µ*g/mL, ab15580) at 4°C and handled by secondary antibodies (Abcam, biotin conjugated goat polyclonal vector, 1 : 250) and observed with a microscope. Mean intensities for positive Ki-67 expression were determined with Image software. Randomly selected the percentage of Ki-67 positive staining from five fields.

### 2.16. Statistical Analysis

Data are measured as mean ± standard deviation. Statistically significant differences were evaluated with one-way or two-way ANOVA or an unpaired student's *t*-test followed by a Tukey's post-test. *P* < 0.05 was statistical significance.

## 3. Results

### 3.1. miR-613 Is Lessened in NSCLC

**Figure 1:**
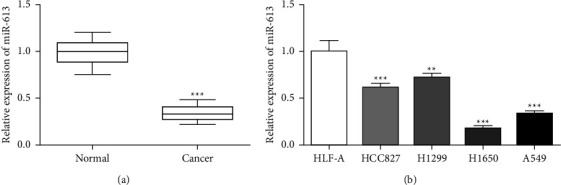
miR-613 expression in non-small cell lung cancer tissues and cells. (a) After harvesting twenty pairs of NSCLC and adjacent normal tissues, the miR-613 expression was tested in NSCLC and control groups using quantitative real-time PCR (qRT-PCR). (b) qRT-PCR analysis for miR-613 expression in human lung cell lines HLF-A and NSCLC cells HCC827, H1299, H1650, and A549. ^*∗∗*^*P* < 0.01*vs.* HLF-A. ^*∗∗∗*^*P* < 0.001* vs.* Normal or HLF-A.

To clarify the miR-613 function in NSCLC, we determined the miR-613 level in NSCLC tissues and found a decreased miR-613 level in NSCLC tissues (Figure 1(a)). Meanwhile, miR-613 dropped in NSCLC cells HCC827, H1299, H1650, and A549 compared with normal human lung cell lines HLF-A, and the decrease was most notable in H1650 cells (Figure 1(b)), which shows that the differential expression of miR-613 is most obvious in H1650 cells. Therefore, we selected H1650 cells for subsequent experiments. In summary, our study confirmed that miR-613 is lowly expressed in NSCLC tissues and cells, which suggests that miR-613 may be a potential therapeutic target for NSCLC.

### 3.2. miR-613 Overexpression Advances NSCLC Cell Apoptosis and Represses Cell Proliferation

**Figure 2:**
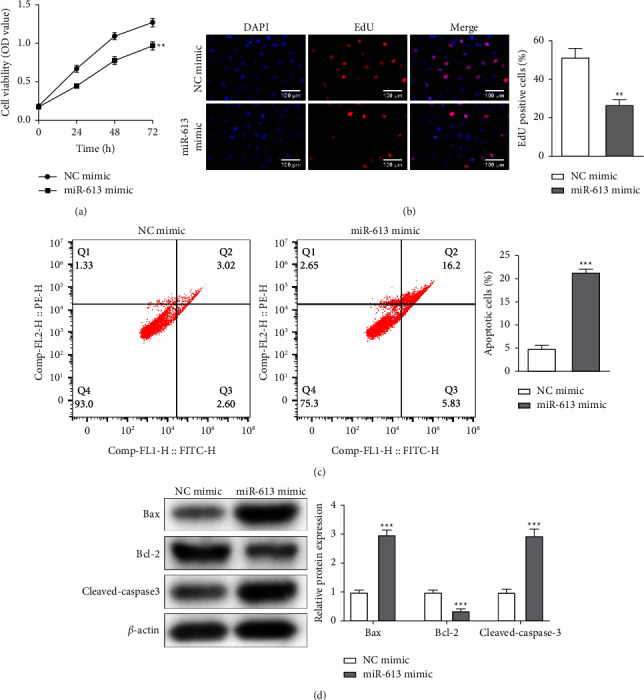
Analysis of miR-613 on NSCLC cell proliferation and apoptosis. miR-613 mimic was transfected into H1650 cells. (a) Evaluation of H1650 cell viability by cell counting kit-8 (CCK-8). (b) Edu method was conducted to assess H1650 cell proliferation (scale bar: 100 *μ*m). (c) H1650 cell apoptosis was determined by flow cytometry. (d) The levels of apoptosis-related proteins Bax, Bcl2, and cleaved caspase-3 were tested by Western blot. ^*∗∗*^*P* < 0.01, ^*∗∗∗*^*P* < 0.001* vs.* NC-mimic. NC: negative control.

Due to the decreased miR-613 in H1650 cells being most remarkable, H1650 cells were selected to verify the miR-613 function in NSCLC development. After transfecting the miR-613 mimic into H1650 cells, CCK-8 results corroborated that the miR-613 overexpression reduced the H1650 cell viability (Figure 2(a)). Meanwhile, the H1650 cell proliferation was weakened after the transfection of the miR-613 mimic (Figure 2(b)). By contraries, flow cytometry analysis expounded that the miR-613 overexpression enhanced the H1650 cell apoptosis (Figure 2(c)). Bax, Bcl-2, and cleaved-caspase-3 were apoptosis-related proteins [26]. As displayed in Figure 2(d), Bax and cleaved-caspase-3 were elevated after miR-613 mimic transfection, while the Bcl-2 was decreased. Overall, the miR-613 overexpression restrained NSCLC cell proliferation and accelerated cell apoptosis.

### 3.3. miR-613 Overexpression Weakens NSCLC Cell Invasion and Migration

**Figure 3:**
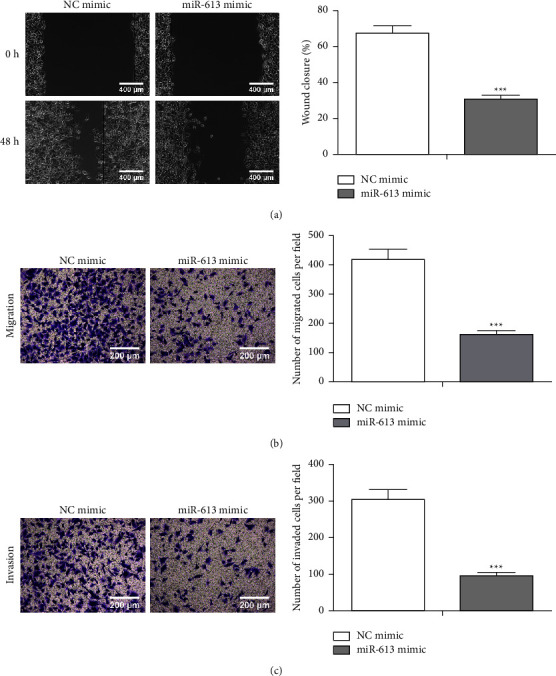
Regulation of miR-613 on NSCLC cell migration and invasion. miR-613 mimic and NC-mimic were transfected into H1650 cells. Wound-healing (a) (scale bar: 400 *μ*m) and Transwell analyses (b) (scale bar: 200 *μ*m) were conducted to evaluate H1650 cell migration. (c) The H1650 cell invasion was assessed using Transwell (scale bar: 200 *μ*m). ^*∗∗∗*^*P* < 0.001*vs. *NC-mimic.

Subsequently, we continued to evaluate miR-613 impact on NSCLC cell invasion and migration. As exhibited in Figure 3(a), miR-613 mimic repressed H1650 cell migration, and this conclusion was further validated by Transwell analysis (Figure 3(b)). Meanwhile, the H1650 cell invasion was restrained after the miR-613 overexpression (Figure 3(c)). To sum up, the miR-613 overexpression restrained NSCLC cell invasion and migration.

### 3.4. miR-613 Represses the M2 Macrophage Polarization

**Figure 4:**
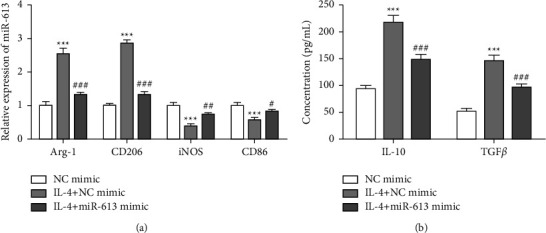
miR-613 regulates the M2 macrophage polarization. After differentiating THP-1 cells into macrophages with 24 h treatment of 150 nM phorbol 12-myristate 13-acetate, the cells were fostered for 48 h with 20 ng/mL IL-4 to achieve the M2 macrophages, and miR-613 mimic was transfected into M2 macrophages. (a) The expressions of Arg-1 and CD206 (markers for M2 macrophages) and iNOS and CD86 (markers for M1 macrophages) were measured using qRT-PCR. (b) The IL-10 and TGF-*β* levels in cell culture supernatant were tested by enzyme-linked immunosorbent assay (ELISA). ^*∗∗∗*^*P* < 0.001* vs.* NC-mimic. ^#^*P* < 0.05, ^##^*P* < 0.01, ^###^*P* < 0.001* vs.* IL-4+NC-mimic.

Previous studies authenticate that M2 macrophage polarization accelerates NSCLC progression [27, 28]. Thus, we attempted to clarify whether miR-613 had an inhibitory effect on M2 macrophage polarization. After THP-1 cells were induced to differentiate into macrophages, a miR-613 mimic was transfected into IL-4-induced M2 macrophages. miR-613 mimic transfection efficiency in M2 macrophages was verified, and miR-613 was lowly expressed in M2 macrophages (Supplementary Figure 1). Arg-1, CD206 are M2 macrophage markers, iNOS, CD86 are M1 macrophage markers [29]. We found that Arg-1 and CD206 were elevated, while iNOS and CD86 were decreased after IL-4 treatment, and these trends were partially reversed by miR-613 mimic transfection (Figure 4(a)).

M2 macrophages usually excrete anti-inflammatory factors such as TGF-*β* and IL-10 [30]. Thus, we further evaluated the TGF-*β* and IL-10 levels in the cell culture supernatant. ELISA results clarified that the IL-10 and TGF-*β* contents were elevated after IL-4 treatment, while miR-613 mimics partially reversed this trend (Figure 4(b)). The abovementioned experimental results illustrated that miR-613 restrained the M2 macrophage polarization.

### 3.5. miR-613 Restrains NSCLC Cell Growth by Reducing M2 Macrophage Polarization

**Figure 5:**
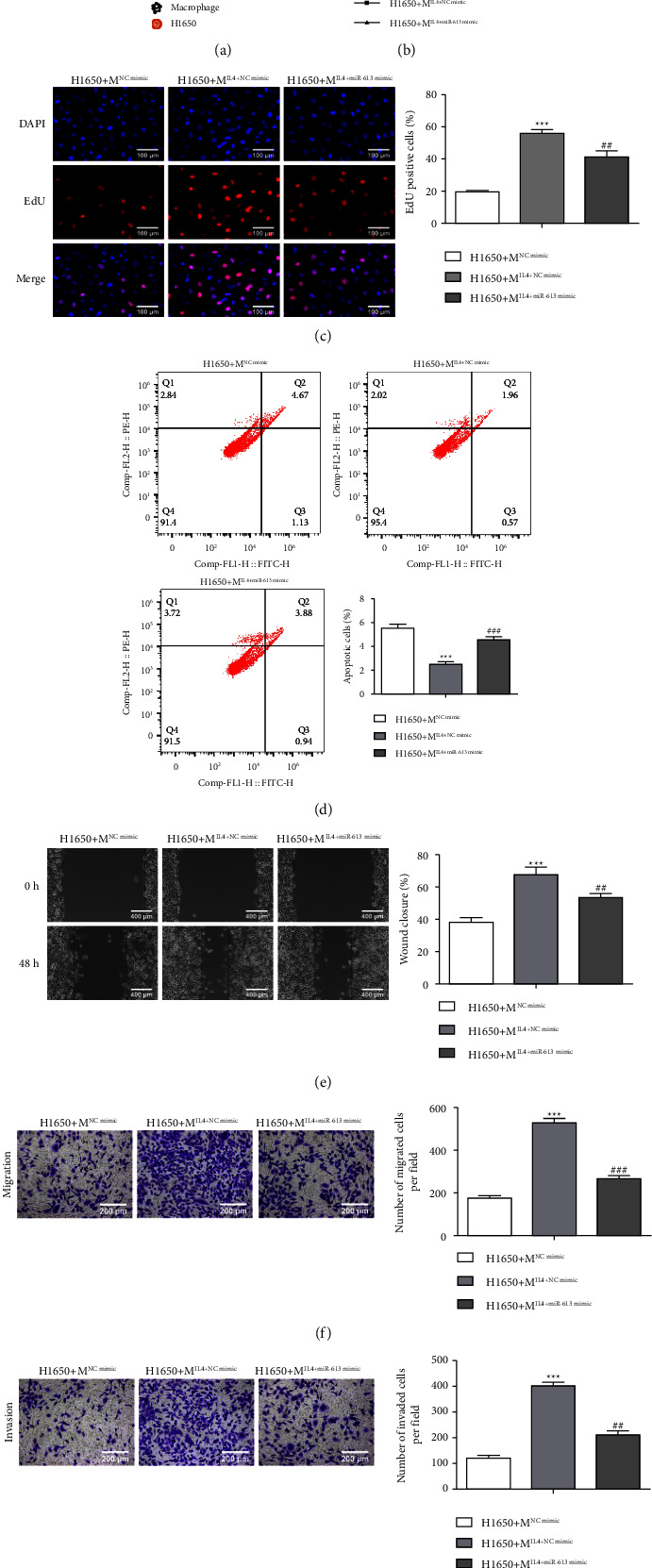
miR-613 regulates NSCLC cell growth by repressing M2 macrophage polarization. Macrophages with different treatments (as in Figure 4) were co-cultured with H1650 cells. H1650 cells were grouped: H1650+M^NCmimic^, H1650+M^IL-4^^+^^NCmimic^ and H1650+M^IL-4+miR-613mimic^. (a) The schematic diagram of the co-culture system. (b) CCK-8 was conducted to assess H1650 cell viability. (c) Evaluation of H1650 cell proliferation by the Edu method (scale bar: 100 *μ*m). (d) Detection of H1650 cell apoptosis by flow cytometry. (e and f) H1650 cell migration was tested using Transwell (scale bar: 200 *μ*m) and wound-healing analyses (scale bar: 400 *μ*m). (g) H1650 cell invasion was measured by Transwell (scale bar: 200 *μ*m). ^*∗∗∗*^*P* < 0.001* vs.* H1650+M^NCmimic^. ^##^*P* < 0.01, ^###^*P* < 0.001*vs.* H1650+M^IL-4^^+^^NCmimic^.

We further determined whether miR-613 regulated the NSCLC cell growth by repressing M2 macrophage polarization. Macrophages with different treatments (as in Figure 4) were co-cultured with H1650 cells.  Figure 5(a) presents a schematic diagram of the co-culture system. CCK-8 assay authenticated that co-culture of M2 macrophages increased H1650 cell viability, while this increase was partially inverted after the miR-613 was overexpressed in M2 macrophages (Figure 5(b)). The same trend was discovered in the H1650 cell proliferation analysis (Figure 5(c)). On the contrary, the co-culture of M2 macrophages weakened H1650 cell apoptosis, while the miR-613 overexpression partially reversed this effect (Figure 5(d)). Meanwhile, the co-culture of M2 macrophages accelerated H1650 cell migration, while this trend was partially inverted after the miR-613 was overexpressed in M2 macrophages, and the Transwell assay further confirmed this finding (Figures 5(e)–5(f)). Moreover, we further evaluated the H1650 cell invasion, and the results expounded that the co-culture of M2 macrophages facilitated H1650 cell invasion, while the miR-613 overexpression partially reversed this effect (Figure 5(g)). In summary, miR-613 restrained NSCLC cell growth by reducing M2 macrophage polarization.

### 3.6. miR-613 Represses Tumor Growth via Restraining M2 Macrophage Polarization *in Vivo*

**Figure 6:**
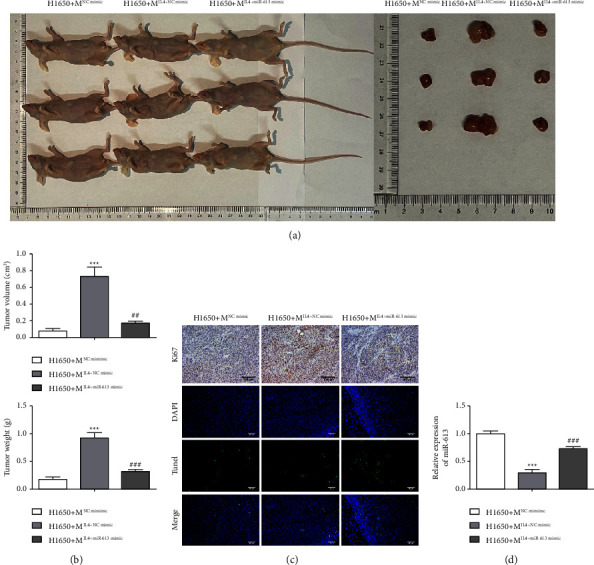
miR-613 influences tumor growth by reducing the M2 macrophage polarization *in vivo*. Macrophages with different treatments (as in Figure 4) were co-cultured with H1650 cells. H1650 cells were grouped: H1650+M^NCmimic^, H1650+M^IL-4^^+^ ^NCmimic^ and H1650+M^IL-4^^+^^miR-613mimic^. The C57BL/6 mice were subcutaneously injected with the above H1650 cells (5 × 10^5^ cells). (a, b) Analysis of the tumor weight. (c) The proliferation and apoptosis were analyzed by Ki-67 staining and TUNEL staining (scale bar: 100 *μ*m). (d) miR-613 expression in tumors by qRT-PCR. ^*∗∗∗*^*P* < 0.001*vs.* H1650+M^NCmimic^. ^##^*P* < 0.01, ^###^*P* < 0.001*vs.* H1650+M^IL-4^^+^^NCmimic^.

We further evaluated the *in vivo* function of miR-613. As displayed in Figures 6(a) and 6(b), the co-culture of M2 macrophages facilitated tumor growth, but this trend was inverted in part after the miR-613 was overexpressed in M2 macrophages. Meanwhile, Ki67 staining confirmed that the co-culture of M2 macrophages accelerated the proliferation, and the miR-613 overexpression partially reversed this trend (Figure 6(c)). On the contrary, TUNEL staining demonstrated that the co-culture of M2 macrophages restrained the apoptosis, while this restraint was partially reversed after the miR-613 was overexpressed in M2 macrophages (Figure 6(c)). Furthermore, miR-613 was decreased after co-culture of M2 macrophages, and this decrease was partially reversed by miR-613 overexpression (Figure 6(d)). These data confirmed miR-613 overexpression restrained tumor growth by repressing M2 macrophage polarization.

## 4. Discussion

The high global mortality rate from NSCLC is a major issue and the challenges of cancer-related treatment [31]. Various miRNAs are pivotal regulatory molecules in NSCLC [7, 32]. Similarly, our study authenticated that miR-613 was lessened in NSCLC. Furthermore, our research demonstrated that miR-613 overexpression restrained NSCLC cell proliferation, migration, invasion, and enhanced apoptosis. Meanwhile, we confirmed that miR-613 overexpression restrained NSCLC growth by repressing M2 macrophage polarization. The completion of this research might provide promising biomarkers for NSCLC treatment.

Accumulated evidence suggests that miR-613 is interrelated to various tumor genesis and development. For instance, miR-613 is lowly expressed in colorectal cancer and has diagnostic and prognostic functions in colorectal cancer [33]; miR-613 represses hepatocellular carcinoma cell dedifferentiation through the SOX9 signaling, which provides novel therapeutic targets for hepatocellular carcinoma [34]. It has been authenticated that miR-613 initiates NSCLC cell cycle arrest via regulating CDK4 [35]. Similarly, we verified that miR-613 was lessened in NSCLC cells and tissues. Simultaneously, we further confirmed that the transfection of miR-613 mimic advanced cell apoptosis and weakened NSCLC cell proliferation, migration, and invasion through various gain-of-function assays, which was similar to a previous conclusion [36].

Previous reports state that macrophages with carcinogenic functions in tumor environments are considered as TAMs and typically exhibit an M2 phenotype [37, 38]. Increasing studies authenticate that M2 macrophages are interrelated to multiple tumors development. For instance, Sousa et al. confirmed that the high number of M2 macrophages is relevant to rapid proliferation and poor prognosis of human primary breast tumors [39]; Yamaguchi et al. clarified that the peritoneal TAMs polarization into M2 phenotype facilitates the gastric cancer tumor growth, prompting that intraperitoneal TAMs is a promising target for gastric cancer [40]. Recently, the pivotal function of M2 macrophage polarization in NSCLC has attracted widespread attention from researchers. M2 macrophages activate the ERK1/2/Fra-1/slug pathway through epithelial-mesenchymal transformation to accelerate the malignant development of NSCLC [41]. Puerarin reduces the M2 macrophage metastasis and polarization of NSCLC transplant-tumor-associated macrophages by inactivating MEK/ERK 1/2 signaling, thereby alleviating NSCLC [42]. Considering the critical functions of miR-613 and M2 macrophages in NSCLC, this study attempted to clarify whether miR-613 alleviated NSCLC by mediating M2 macrophage polarization. As expected, our experimental data revealed that miR-613 restrained NSCLC growth through repressing M2 macrophage polarization.

However, there are still some limitations in this study, and although, we found that miR-613 regulated NSCLC development by inhibiting M2 macrophage polarization, whether miR-613 would regulate NSCLC development by regulating downstream gene targets or oxidative stress response to inhibit M2 macrophage polarization still needs further investigation.

In general, we confirmed miR-613 overexpression enhanced apoptosis and restrained NSCLC cell proliferation, migration, and invasion. Meanwhile, miR-613 overexpression restrained NSCLC growth by repressing M2 macrophage polarization, which provides novel insights and strategies for treating NSCLC.

## Figures and Tables

**Table 1:** Primer sequence used in qRT-PCR.

Gene name	Primer sequence (5′-3′)
miR-613	Forward: GTGAGTGCGTTTCCAAGTGT
	Reverse: TGAGTGGCAAAGAAGGAACAT

Arg-1	Forward: AGGCGCTGTCATCGATTTCT
	Reverse: TGGAGTCCAGCAGACTCAAT

CD206	Forward: CTCTGTTCAGCTATTGGACGC
	Reverse: CGGAATTTCTGGGATTCAGCTTC

iNOS	Forward: GCGCTCTAGTGAAGCAAAGC
	Reverse: AGTGAAATCCGATGTGGCCT

CD86	Forward: CTTTGCTTCTCTGCTGCTGT
	Reverse: GGCCATCACAAAGAGAATGTTAC

*β*-actin	Forward: CTCCATCCTGGCCTCGCTGT
	Reverse: GCTGTCACCTTCACCGTTCC

U6	Forward: CTCGCTTCGGCAGCACA
	Reverse: AACGCTTCACGAATTTGCGT

## Data Availability

The data used to support the findings of this study of the study are available from the corresponding author upon reasonable request.

## Supplementary Materials

Supplementary Materials Supplementary Figure 1 Analysis of the miR-613 expression in M2 macrophages. After THP-1 cells were treated with 150 nM PMA for 24 h to be differentiated into macrophages, the cells were incubated with 20 ng/mL IL-4 for 48 h to achieve M2 macrophages, and miR-613 mimic was transfected into M2 macrophages. The miR-613 expression in M2 macrophages was determined using qRT-PCR. ^*∗∗∗*^*P* < 0.001 vs. control. ^###^*P* < 0.001 vs. IL-4+NC-mimic.
