# Supplementary material for: Tumor Suppressor miR-613 Alleviates Non-Small Cell Lung Cancer Cell via Repressing M2 Macrophage Polarization
Source: J Oncol. 2023 Feb 16;2023:2311231. doi: 10.1155/2023/2311231 (PMC9950322; doi:10.1155/2023/2311231)
Supplement: Supplementary Materials — Supplementary Figure 1 Analysis of the miR-613 expression in M2 macrophages. After THP-1 cells were treated with 150 nM PMA for 24 h to be differentiated into macrophages, the cells were incubated with 20 ng/mL IL-4 for 48 h to achieve M2 macrophages, and miR-613 mimic was transfected into M2 macrophages. The miR-613 expression in M2 macrophages was determined using qRT-PCR. ∗∗∗P < 0.001 vs. control. ###P < 0.001 vs. IL-4+NC-mimic. [file 2311231.f1.docx]

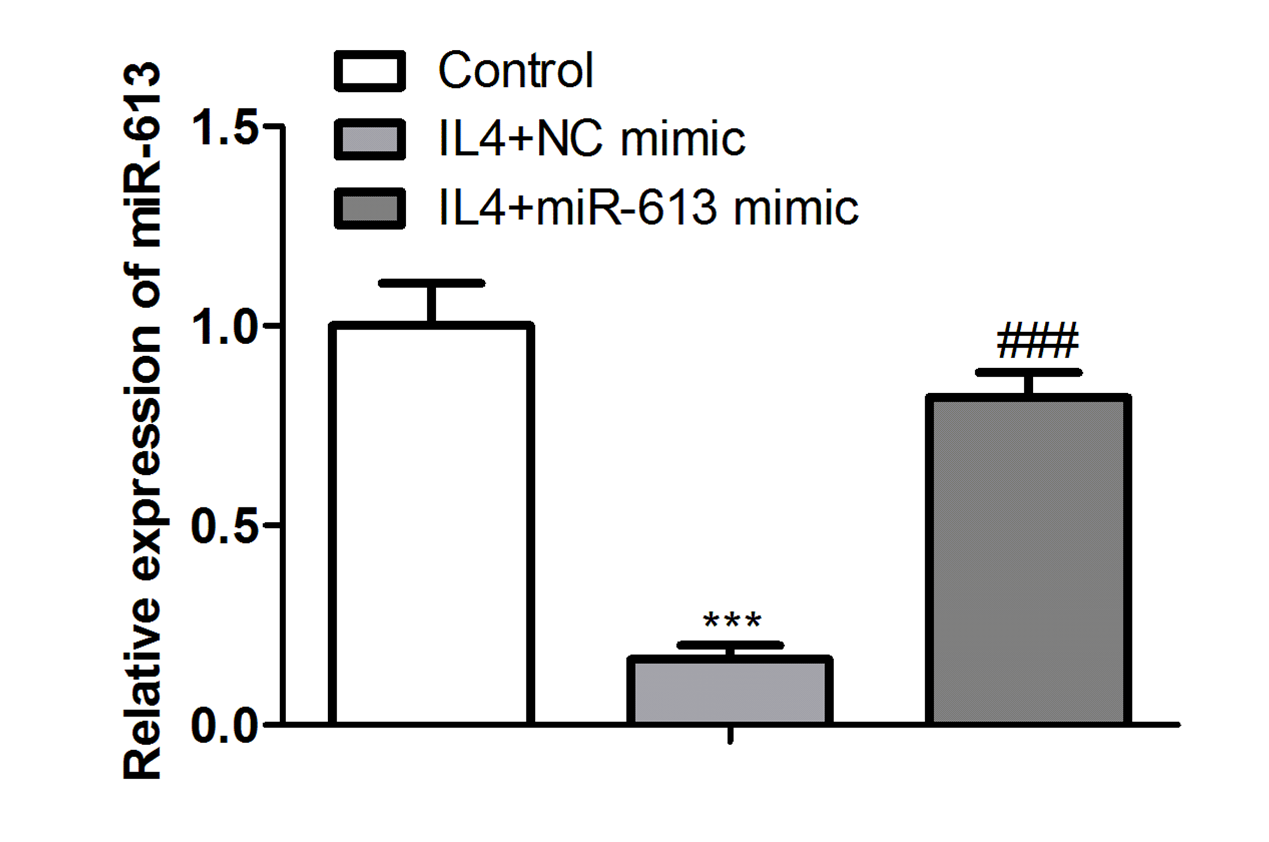


**Supplementary Figure 1 Analysis of the miR-613 expression in M2 macrophages.** After THP-1 cells were treated with 150 nM PMA for 24 h to be differentiated into macrophages, the cells were incubated with 20 ng/mL IL-4 for 48 h to achieve M2 macrophages, and miR-613 mimic was transfected into M2 macrophages. The miR-613 expression in M2 macrophages was determined using qRT-PCR. ****P*<0.001 vs. control. ^###^*P*<0.001 vs. IL-4 + NC-mimic.
